# Transference-focused psychotherapy as an aid to learning psychodynamic psychotherapy: qualitative analysis of UK psychiatry trainees’ views

**DOI:** 10.1192/bjb.2020.129

**Published:** 2022-02

**Authors:** Orestis Kanter Bax, Georgios Nerantzis, Tennyson Lee

**Affiliations:** 1 Deancross Personality Disorder Service, London, UK; 2 Centre for the Understanding of Personality Disorder, London, UK; 3 Essex Partnership University Trust, UK

**Keywords:** Transference-focused psychotherapy, residency, psychodynamic psychotherapy, personality disorders, education and training

## Abstract

**Aims & method:**

Learning psychotherapy can be difficult and stressful. We explore core trainees’ (*n* = 5) views on undertaking a psychodynamic psychotherapy training case using transference-focused psychotherapy (TFP), in an East London NHS Foundation Trust supervision group. We used framework analysis of focus group interviews to examine trainees’ concerns, their views about this experience and its impact on general psychiatric practice.

**Results:**

Trainees described various concerns on starting: providing an effective intervention, insufficient experience and training-related pressures. However, they found that TFP addressed some of them and was helpful for learning psychodynamic psychotherapy. Difficulties around the countertransference remained at end-point. Trainees suggested that introductory teaching and learning through observation might be worthwhile.

**Clinical implications:**

Trainees’ experience suggests that an evidence-based operationalised approach such as TFP can be integrated into the core psychiatry curriculum as a psychodynamic psychotherapy learning method. Trainees report benefits extending to other areas of their practice.

Transference-focused psychotherapy (TFP) is an evidence-based, manualised^1^ treatment for borderline personality disorder,^2–4^ with modifications for narcissistic personality disorder.^5,6^ Developed from Otto Kernberg's contributions on borderline personality organisation,^7,8^ it offers a structured approach to the treatment of severe personality disorders.

TFP offers operationalised technical guidance based on modified psychoanalytic principles that are helpful for the general psychiatrist.^9^ We hypothesised that there are specific TFP characteristics (Box 1) that may be facilitators for a meaningful training experience in the UK, especially in the context of training in the National Health Service (NHS) and the drive for developing suitable skills for a patient population with severe personality pathology. TFP has been used in psychiatric residency training in Australia and the USA with positive learning outcomes for residents.^10–12^ It has recently been introduced in the UK as a modality for the supervised experience of trainees and this is the first reported study describing trainees’ views.

Box 1 About transference-focused psychotherapy

Transference-focused psychotherapy (TFP) is a psychodynamic treatment grounded in contemporary object relations theory and supported by randomised controlled trials. Its main premise is the central importance of transference analysis, as manifested in the ‘here and now’ of the therapeutic relationship, in relation to the integration of fragmented psychological structures (borderline personality organisation).

The treatment is operationalised in a treatment ‘manual’^1^ that provides the technical framework (strategies, tactics and techniques) to be used by the therapist. Key modifications from traditional psychoanalysis include an emphasis on a diagnostic ‘structural assessment’, lesser frequency (1–2 sessions/week), more active participation by the therapist, and focus on the treatment frame and treatment objectives as negotiated in the treatment contract (with limit setting when self-destructive behaviours occur).

In line with its psychoanalytic origin, TFP examines various communications at different levels (verbal, non-verbal, countertransference) and aims to help the patient understand and resolve unconscious conflicts and work towards greater personality integration.

## Background

The role of psychodynamics in medical education and psychiatric training curricula dates from the beginning of psychoanalysis.^13^ Tensions between the medical model in psychiatry and the psychoanalytic paradigm remain.^14^ The Royal College of Psychiatrists (RCPsych) places importance on psychotherapy learning in core and higher psychiatry training in the UK.^15^ Core trainees need to meet psychotherapy-specific curriculum competencies: participation in Balint-type or case-based discussion groups and supervised psychotherapy experience. Completion of core training and examinations lead to RCPsych membership and eligibility for progression to higher specialty training. The aim is not for all trainees to progress to medical psychotherapy specialism, but for psychiatrists to be psychotherapeutically informed.^16^

Trainees’ views on case-based discussion groups^17^ and cognitive–behavioural therapy (CBT)^18^ indicate that these are valued as part of their training development. Psychodynamic psychotherapy has been used for learning psychodynamic principles and for the long-case requirement.^19^ Reports indicate that starting out in psychodynamic psychotherapy is daunting^20^ and related ideas are challenging but valuable.^21,22^ The General Medical Council's Medical Psychotherapy Report (2013) has triggered further research on psychotherapy training needs. There is accumulating evidence about specific specialist approaches and their usefulness for the trainee psychiatrist.^23,24^

## Aims

This study was set up to identify the baseline and end-of-therapy views of core trainee psychiatrists in East London NHS Foundation Trust (ELFT) regarding a TFP approach to their psychodynamic long case. We investigated (a) concerns about starting out, (b) recommendations regarding training needs, (c) the perceived advantages and disadvantages of using TFP and (d) its impact on current and future psychiatric practice.

## Method

Five core trainees in the same supervision group were identified and invited to participate in the study. They were informed that participation was voluntary, that the interviewer of the focus group discussions would be independent from their supervisory structure and that transcripts would be anonymised before analysis.

We conducted two focus group interviews with the participants, one at the start and one at the end-point of their training case. We employed a framework approach^25–27^ owing to its suitability for analysis of textual data, particularly semi-structed interview transcripts. The focus group interviews were facilitated by a clinical psychologist with no background in TFP. The interviewer asked five questions designed to investigate the study's initial aims. Two were asked a baseline:

- What are your main concerns about starting a psychodynamic long case?
- What would you find most helpful in starting a psychodynamic long case?

and the remaining three at end-point:

1. What are the advantages of using TFP for your first psychodynamic long case?
2. What are the disadvantages of using TFP for your first psychodynamic long case?
3. How has the experience of having used TFP on a psychodynamic long case affected your current and ongoing psychiatric practice?

Open, non-suggestive prompts repeating the question and inviting further elaboration or participant dialogue, and a prompt at end-point inviting trainees to think back to their baseline concerns, were permitted. Discussions were audio recorded, lasting 75 and 60 min each. We transcribed recordings verbatim and anonymised transcripts ahead of independent familiarisation with the data by the interviewer and the lead author. Line-by-line open coding was conducted independently by the two, who subsequently compared labels, resolved differences through discussion, homogenised themes and jointly produced the resulting indexes and charts. Additional deductive review of the transcripts, mapping of themes, analysis and interpretation were completed jointly in a final stage by two of the authors and the interviewer.

All participants (in year 2 and year 3 of training at baseline), two males and three females, provided consent to the study. All had already completed the academic and basic clinical part of their training for their level (psychotherapy lectures, case-based discussion groups) and all but one had completed their CBT short case. None had formal psychodynamic psychotherapy experience or had worked in a personality disorder service and all were expected to undertake preparation for the training method (Table 1). All joined the baseline focus group interview and one did not take part in the end-point interview. All met their training expectations, and all except one (the patient discontinued psychotherapy early) completed their case after 1 year.

**Table 1:** Characteristics of the transference-focused psychotherapy (TFP) training method

Characteristics	Details
Format of clinical experience	Individual TFP once a week
Duration	1 year
Allocated patients’ characteristics	Personality disorder diagnosis: borderline personality disorder or narcissistic personality disorder (as assessed on SCID-II DSM-IV).^28^ Moderate to severe pathology. Informed consent for session audio-recording of all sessions for training purposes.
Patient preparation requirements	Assessment, preliminary contract setting^29^ and goal setting completed by the specialist service. Trainee-led contract finalisation takes place in a preliminary meeting after allocation.
Trainee preparation requirements	Familiarisation with the TFP ‘manual’.^1^ Orientation to the supervision process and regular preliminary attendance of the group.
Supervision characteristics	Facilitated by a consultant psychiatrist in medical psychotherapy and accredited TFP teacher. Format: group. Frequency: once weekly. Duration: 1 h. 1:1 preparation and progress review meetings with the supervisor at start, midpoint and end of case. Review and supervision of audio-recorded sessions. Review of end-of-case formulation report

Cases related to this study were assessed prior to allocation and consideration was given to reducing the risk of patient drop-out. Patients allocated had borderline personality disorder or narcissistic personality disorder diagnoses and scored in the lower range of the Global Assessment of Functioning (GAF) (scores of 51–10), indicating serious impairment.

The study was registered as a service development project and was granted approval by the East London NHS Foundation Trust Ethics Committee.

## Results

Major themes from our analysis matched the questions asked. The subthemes derived from the data, and we present findings in two sections, each following analysis of the respective interviews. The first section (Trainees’ concerns) addresses the first two aims of our study using baseline data (Table 2) and the second (Trainees’ views) the last two, with end-point data (Table 3).

**Table 2:** Thematic analysis: focus group (5 participants) views at baseline

Themes	Subthemes
(1) Concerns about taking on a long case	Providing an effective intervention Insufficient experience and training Training-related and other pressures
(2) Recommendations about taking on a long case	Practical training skills Improvement in supervision Introductory teaching Common scenarios and basic tips Access to personal psychotherapy

**Table 3:** Thematic analysis: focus group (4 participants) views at end-point

Themes	Subthemes
(1) Advantages of using TFP	Clarity about the nature of psychotherapy Improved ability to manage the therapeutic encounter Facilitates long-case supervision Facilitates long-case preparation Facilitates learning psychodynamics
(2) Disadvantages of using TFP	General/unspecified limitations Limitations related to countertransference management Difference from the psychiatric model
(3) Impact on initial concerns about taking on a long case	Reduces anxiety about doing it wrong Reduces anxiety about doing it right Reduces anxiety about the patient discontinuing psychotherapy early Reduces anxiety about making interventions Facilitates formulating/planning treatment No effect on concern about lack of effectiveness Positive effect on concern about lack of effectiveness No effect on concern about training commitments
(4) Effects on psychiatric practice	Positive effect on the clinical encounter Positive effect on understanding of personality disorder Positive effect on working in team Positive effect on education and training skills

TFP, transference-focused psychotherapy.

### Trainees’ concerns about starting a psychotherapy long case and their recommendations

**‘**I have never even seen any kind of talking therapy happen […] normally in medicine you sort of at least see something, like someone put in a cannula or someone take a history’ (participant 1).

**‘**[…] that's probably quite difficult for us to swallow, being medical, ’cause I think you always think there needs to be some sort of result, whereas maybe there isn't always some big result that you want, but in our heads we probably think that every time there should be, so this puts more pressure on you’ (participant 3).

Trainees expressed concerns at baseline about treatment effectiveness and lack of competency: ‘the intervention I am doing, how therapeutic is it?’ (participant 5); ‘is it a waste of time?’ (participant 1). They said they had no previous training and experience, no direct observation of treatment delivery, a limited theoretical and conceptual map and limited familiarity with the field: ‘you don't even know how you are supposed to sit’ (participant 1); ‘I don't have that deep knowledge of psychoanalysis to really understand what I'm doing’ (participant 2). This left them with uncertainty, a sense of lack of purpose, inadequacy and unpreparedness. They were concerned about the quality of care they delivered, potential errors in treatment delivery, negative treatment effects and causing harm to the patient: ‘I am a bit worried about saying the wrong thing and sort of causing damage, I guess people who have actually trained in therapy would be less likely to’ (participant 3); ‘the feel of failure is quite strong: is it gonna be your fault?’ (participant 5).

Additional pressures originated from their wish to complete their long case in a timely manner for training progression: ‘one of the big concerns is about the [patient] dropping out before doing enough work to complete the case’ (participant 2). They discussed the emotional and personal commitments to the patient and the task in hand, and reported pressures relating to the continuity and intensity of contact and a sense of isolation. Some trainees mentioned having had no personal psychotherapy as an added concern.

Given perceived limitations in the current format for preparation for the long case and the limited duration of supervision sessions, trainees recommended introductory teaching (theory and technique) and suggested focusing on practical skills and observational learning (audio, video, simulation and expert demonstration methods): ‘seeing someone having psychodynamic work in practice’ (participant 2); ‘a few key [tips]: tell me in three sentences what am I supposed to be doing when I start’ (participant 1). They also noted that anxiety management skills would be useful.

### Trainees views on learning psychodynamic psychotherapy using TFP and its impact on psychiatric practice

**‘**I think I was really worried that I was going to do it [psychotherapy] wrong or not be able to do it or not know what I was doing […] I think the more you do it you realise there isn't really a right and wrong […] I think you have a lot more anxiety about it before you start’ (participant 3).

**‘**at the beginning [I didn't] see the point of a trainee doing a long case if you have no interest in going into doing psychotherapy training as an SpR [specialist registrar], but I think it has changed my clinical practice […] I would hope it's not something I would forget or lose as I go through the career’ (participant 4).

‘you get a sort of more rounded view of how they [patients with personality disorder] feel, I think that they suffer more […] and I understand what it's like for them a bit more’ (participant 1).

At the point of completion of the long case, trainees discussed the positives and negatives of using TFP, its impact on their initial concerns and day-to-day psychiatric practice.

They reported an overall positive effect on their initial concerns: TFP reduced anxiety about competence, harming the patient, the patient discontinuing psychotherapy early and making therapeutic interventions. They remained worried about the effectiveness of psychotherapy. There was no impact on their concerns about competing training pressures.

Trainees said that TFP provided clarity about the nature and purpose of psychotherapy and it made the theory more accessible and less obscure. They also spoke about TFP enabling a focus on the patient–therapist relationship ‘in the here and now’, and their increased ability to focus on affect, challenge engagement on a cognitive level, address recurrent transference and countertransference patterns and manage the negative transference: ‘I think it is clearer what you are supposed to be doing with TFP’ (participant 4); ‘It helps you mentalise yourself a bit more in the session’ (participant 3); ‘with TFP alone I guess you are quite protected in some way, because however [the patients] respond even if it is quite negative, this could be a positive thing, ’cause there was a lot of affect in the room, there is a lot of material’ (participant 3).

They reported that the treatment contract and frame provided a shared sense of purpose for themselves and the patient, reduced their anxiety about interventions and activity in the session, and enabled them to manage risk, address acting out and better understand the patient's expectations: ‘I think the TFP frame was useful in […] thinking about why that happened without it feeling really personal’ (participant 2); ‘in TFP you are more allowed to bring up breaks to the contract – with my patient I felt much more comfortable to do this’ (participant 4).

Finally, they shared a view that TFP enables preparation at baseline and ahead of each session, allowing them to recall sessions and report them in supervision in a structured way. They said it facilitated the use of supervision within time constraints by offering a shared language and reference framework, which allowed them to track affectively important material, understand and feedback in supervision the challenges to the frame and the patient–therapist relationship: ‘[TFP] framed how I would relate the session back to the group’ (participant 2).

In terms of the shortcomings of TFP, they felt that its focus on the transference neglected other important relationships in the patient's life and limited the variety of potential directions for the therapy. They reported that the expectation of therapist activity and the manualised model produced performance and adherence anxiety: ‘I was quite anxious at least for the first 10 sessions to make sure I was on model’ (participant 2). Some trainees said that TFP theory and practice was difficult to link and that the marked difference from the psychiatric model, in combination with their limited exposure to psychoanalytic theory, was a limitation for using the model. They said that the challenges in managing countertransference-related difficulties were not fully overcome by using TFP. They described negative therapist feelings produced by the focus on the transference, and difficulties with maintaining therapeutic neutrality. They also mentioned experiencing uncertainty in the face of patients developing positive feelings, attachment to the therapist, genuine affective contact and psychological progress: ‘I feel like my patient has made progress and that he is being genuine and the more he is like that, the more difficult it is for me to know what to do’ (participant 3).

Trainees noted that TFP experience improved their daily psychiatric practice and working with patients in various general adult psychiatric settings: ‘I find it easier to be clear about the point of us meeting and to maintain the boundaries around that, whereas before when I first started I always found it hard to keep my clinic sessions down to the right length [and to] be clear with them and myself about why we are meeting’ (participant 2). They felt it improved awareness and management of transference/countertransference ‘in the room’ in such settings, made interactions with patients (especially those with a personality disorder) less stressful and improved their ability to manage boundaries, set therapeutic goals and contracts, promote openness, instil hope, manage time, risk and acting-out, and liaise with specialist services. They felt that TFP improved their understanding of the nature of personality disorder and the patients’ subjective experience: ‘It gives you a better hope’ (participant 1); ‘I think you just have […] more understanding of it [personality disorder]’ (participant 3); ‘I think I am more confident […] managing assessments or interactions […] being boundaried and also commenting on things that I might not know how to comment on before and being quite open with the patient in a professional way’ (participant 3).

They said that working in teams was positively affected through an improved ability to supervise teams, and to recognise and address colleagues’ and teams’ strong affective reactions to interactions with patients with personality disorders: ‘you are able to have that discussion [about frustration and acting out] with your colleagues, like PLNs [psychiatric liaison nurses]’ (participant 2); ‘using the TFP sort of structure [to think] about actually why is this anxiety being raised, why is the team acting in such a way’ (participant 4). They reported that their experience provided transferable skills for tutoring and education and improved their understanding of the role of psychotherapy in the curriculum.

Two participants said this experience motivated them to seek further experience in psychotherapy.

## Discussion

Trainees with no previous experience in psychodynamic psychotherapy expressed intense anxieties related to the prospect of providing this intervention for the first time. They described ambivalence about the value of psychodynamic psychotherapy at baseline, professional self-doubt and training-related pressures. The interview after completion of their long cases indicated that some of these anxieties are alleviated and that TFP has overall positive effects for trainees, extending into their general psychiatric practice, though with some limitations.

Describing therapist difficulties is a core area of the psychotherapy literature but there are few publications specifically identifying what psychiatry trainees find difficult.^20,30,31^ Our study group's anxieties partly match the available taxonomy for psychotherapists’ (both novice and seasoned) struggles.^32^ This pattern of self-doubt, recognised for the novice therapist,^33^ is to be expected also for the trainee psychiatrist in the early stages of development but may improve with professional progression.^34^ This trajectory of change is supported by our findings of some improved anxieties at end-point. Trainees report increased confidence about doing psychotherapy and working with patients with personality disorders in the general psychiatric setting.

Manualisation in psychodynamic psychotherapy is an emerging trend.^35^ Trainees hold contrasting views about this aspect of TFP. On the one hand, they reported that the specific treatment framework promoted learning psychodynamic principles in a structured manner and facilitated supervision. On the other hand, they said it produced performance anxieties. Importantly though, trainees reported that it helped with management of risk and acting out. There are limitations relating to persistent scepticism about the effectiveness of psychotherapy, ongoing difficulties in managing the countertransference, TFP's psychoanalytic origin, which trainees feel unfamiliar with and consider at odds with the established medical model, and training-related pressures. It is noteworthy that trainees felt helped in managing their countertransferential feelings in psychiatric settings rather than in their psychotherapy work. This both indicates the complexity of use of the countertransference in psychotherapy and suggests the potential contribution of a psychoanalytically informed training such as TFP for psychiatrists in their daily work.

Evidence indicates a high rate of early dropout in patients receiving psychotherapy.^36^ This is less prevalent in psychiatric training^37^ but important to consider, given the impact on training progression. TFP was reported to affect concerns about doing psychotherapy, but it did not affect the experience of training-related pressures.

Given the increasing complexity of the population seen in secondary care, finding suitable training cases may be difficult. Cases treated in this study had moderate to severe personality disorder. TFP may facilitate a pragmatic approach to training within the current NHS service limitations. Four of the five trainees were able to complete their cases and described making good use of the intervention. Supervisors may wish to consider trainees’ competence when allocating cases^38^ and be aware of the difficulties relating to the focus on the transference, trainees’ performance anxieties, pressures about ‘being on model’ and the lack of access to personal therapy.^39,40^ Trainees reported that using a TFP approach partially reduced levels of anxiety about doing psychotherapy with a subgroup of patients with complex problems.

Supplementing supervision with other modes of preparation and learning, with a focus on direct observation, audio-visual material^41,42^ and simulated learning should be considered for incorporation into standard supervision groups.

### Strengths and limitations

This is the first study in the UK to examine the views of core trainees about doing a long psychotherapy case using TFP as the modality of choice. TFP has been recently introduced in the country and its use for training purposes in the NHS only recently started. We provide an early description of trainees’ views about this evidence-based treatment, linking it with training-specific needs.

Limitations in this study are inherent to the study design and the small number of participants, typical of the size of supervision groups in core training. Theoretical saturation in the analysis may be suboptimal because of the small sample size,^43^ but limited use of TFP in the UK for training purposes did not allow the inclusion of further focus groups at this time. We studied the views of trainees in a single supervision group, in one locality within one NHS trust. The preparation and supervision were specific to this locality and the generalisability of findings is limited by this. We anticipate that as use of the model grows in the UK, opportunities will arise for further studies of trainees’ views that may in addition explore patients’ experience of TFP.

We safeguarded against positive bias by explicitly separating the training evaluation process from participation in the study from the outset. Participation in the study was optional. The long-case supervisor did not facilitate the focus group discussions, and the transcripts were anonymised to limit the supervisor's ability to identify a specific trainee's comments and views and to limit self-censorship and selective reporting. The participants were explicitly invited to report their views about the negatives of using TFP to further mitigate this risk and they indeed shared a range of opinions. The risk that the focus group responses may have been biased by the participants’ perceived expectations of their supervisor is nevertheless present. Attrition at end-point (one trainee did not participate in the end-point focus group) also reinforces the risk of positive bias in the results presented.

### Recommendations

Findings from our study of trainees’ views about using TFP support the use of this model for core training purposes. Learning psychodynamic psychotherapy can be difficult and stressful, and this model of training delivery addresses some of the concerns of the starting trainees. We recommend that the use of TFP can help improve their confidence about the effectiveness of psychodynamic psychotherapy and their capacity to treat patients and manage clinical encounters, and can facilitate their use of supervision. There are additional reported collateral benefits for the developing psychiatrist, in terms of working with patients with personality disorders, understanding of psychodynamic aspects in psychiatry, working in teams, and improving education and training skills.

Learning psychodynamic psychotherapy remains a fundamentally challenging endeavour that requires working with and tolerating uncertainty. The problem of some trainees experiencing a dissonance between established psychiatric training and the psychoanalytic principles used by TFP remains, and further integration between disciplines is still required.^44^ TFP does not offer a magic bullet for the intrinsically complex nature of learning psychodynamic psychotherapy. However, our study suggests that it addresses some of the trainees’ anxieties about taking this task on.

On the basis of these findings, we recommend that the RCPsych considers using TFP in its core training curriculum for meeting the psychotherapy long-case requirement. Our findings are in keeping with evidence from the international paradigm about the usefulness of TFP for psychiatry training purposes.

## Data Availability

The data that support the findings of this study are not available to share due to ethical considerations about the privacy of the participants.

## About the authors

**Orestis Kanter Bax** is a Senior Trainee in Adult Psychiatry and Medical Psychotherapy with Deancross Personality Disorder Service (East London NHS Foundation Trust) and Essex Partnership University NHS Foundation Trust. He is also a research associate at the Centre for the Understanding of Personality Disorder (CUSP), London, UK. **Georgios Nerantzis** is a Senior Trainee in Adult Psychiatry currently training at East London NHS Foundation Trust and a reasearch associate at CUSP, London, UK. **Tennyson Lee** is a consultant psychiatrist in medical psychotherapy, clinical lead at Deancross Personality Disorder Service (East London NHS Foundation Trust) and co-director of CUSP, London, UK.

## References

[1] Yeomans F, Clarkin J, Kernberg O. Transference-Focused Psychotherapy for Borderline Personality Disorder: A Clinical Guide. American Psychiatric Publishing, 2015.

[2] Clarkin JF, Levy KN, Lenzenweger M-F, Kernberg OF. Evaluating three treatments for borderline personality disorder: a multiwave study. Am J Psych 2007; 164: 922–8. 10.1176/ajp.2007.164.6.922 17541052

[3] Doering S, Hörz S, Rentrop M, Fischer-Kern M, Schuster P, Benecke C, Transference-focused psychotherapy v. treatment by community psychotherapists for borderline personality disorder: randomised controlled trial. Br J Psychiatry 2010; 196: 389–95. 20435966 10.1192/bjp.bp.109.070177

[4] Levy KN, Meehan KB, Kelly KM, Reynoso JS, Weber M, Clarkin JF, Change in attachment patterns and reflective function in a randomized control trial of transference-focused psychotherapy for borderline personality disorder. J Consult Clin Psychol 2006; 74: 1027–40. 17154733 10.1037/0022-006X.74.6.1027

[5] Diamond D, Hersh R. Transference-focused psychotherapy for narcissistic personality disorder: an object relations approach. J Pers Disord 2020; 34(suppl): 159–76. 32186988 10.1521/pedi.2020.34.supp.159

[6] Diamond D, Yeomans F, Stern B, Levy K, Hörz S, Doering S, Transference focused psychotherapy for patients with comorbid narcissistic and borderline personality disorder. Psychoanal Inq 2013; 33: 527–51.

[7] Kernberg O. Borderline Conditions and Pathological Narcissism. Aronson, 1975.

[8] Kernberg O. Severe Personality Disorders: Psychotherapeutic Strategies. Yale University Press, 1984.

[9] Lee T, Hersh RG. Managing the clinical encounter with patients with borderline personality disorder in a general psychiatry setting: key contributions from transference-focused psychotherapy. BJPsych Adv 2019; 25: 229–36.

[10] Zerbo E, Cohen S, Bielska W, Caligor E. Transference-focused psychotherapy in the general psychiatry residency: a useful and applicable model for residents in acute clinical settings. Psychodyn Psychiatry 2013; 41: 164–81. 10.1521/pdps.2013.41.1.163 23480166

[11] Bernstein J, Zimmerman M, Auchincloss EL. Transference-focused psychotherapy training during residency: an aide to learning psychodynamic psychotherapy. Psychodyn Psychiatry 2015; 43: 201–21. 26039228 10.1521/pdps.2015.43.2.201

[12] Martin L, Lloyd B, Cammell P, Yeomans F. Transference-focused psychotherapy in Australian psychiatric training and practice. Australasian Psychiatry 2017; 25: 233–5. 27679630 10.1177/1039856216671661

[13] Freud S. On the teaching of psycho-analysis in universities. Reprinted in theStandard Edition of the Complete Psychological Works of Sigmund Freud, Vol. 17 (1917–1919): An Infantile Neurosis and Other Works (trans & ed J Strachey). Vintage, 2001.

[14] Wolpert L, Fonagy P. There is no place for the psychoanalytic case report in the *British Journal of Psychiatry*. Br J Psychiatry 2009; 195: 483–7. 19949194 10.1192/bjp.bp.109.064451

[15] Royal College of Psychiatrists. A Competency Based Curriculum for Specialist Core Training in Psychiatry: Core Training in Psychiatry CT1–CT3. RCPsych, 2013.

[16] Johnston J. Learning from the Cradle to the Grave: The Psychotherapeutic Development of Doctors from Beginning to End of s Career In Medicine snd Psychiatry (RCPsych Occasional Paper OP102). RCPsych, 2017.

[17] Das A, Egleston P, El-Sayeh H, Middlemost M, Pal N, Williamson L. Trainees’ experiences of a Balint group. Psychiatr Bull 2003; 27: 274–5.

[18] Carson AA, Clark SE. Psychiatry trainees’ experiences of cognitive–behavioural therapy training in a UK deanery: a qualitative analysis. BJPsych Bull 2017; 41: 97–102. 28400968 10.1192/pb.bp.115.051565 PMC5376726

[19] Johnston J. Royal College of Psychiatrists, The UK Psychotherapy Training Survey. RCPsych, 2013.

[20] Wilson J. Starting out in psychodynamic psychotherapy. Psychiatr Bull 2001; 25: 72–4.

[21] Green J. The training value of psychodynamic psychotherapy long case: a psychiatry trainee's perspective. Br J Psychother 2012; 28: 374–81.

[22] Allison L. Psychoanalytic ideas and their place in psychiatry training in the UK. Lancet Psychiatry 2014; 1: 242–4. 26360735 10.1016/S2215-0366(14)70262-7

[23] Bernanke J, McCommon B. Training in good psychiatric management for borderline personality disorder in residency: an aide to learning supportive psychotherapy for challenging-to-treat patients. Psychodyn Psychiatry 2018; 46: 181–200. 29809114 10.1521/pdps.2018.46.2.181

[24] Brodsky BS, Cabaniss DL, Arbuckle M, Oquendo MA, Stanley B. teaching dialectical behavior therapy to psychiatry residents: the Columbia Psychiatry Residency DBT curriculum. Acad Psychiatry 2017; 41: 10–5. 27481266 10.1007/s40596-016-0593-0 PMC5247344

[25] Pope P, Mays N (eds) Qualitative Research in Health Care (3rd edn). BMJ Books, 2006.

[26] Richie J, Spencer L. Qualitative data analysis for applied policy research. In Analyzing Qualitative Data (eds A Bryman, RG Burgess): 173–94. Routledge, 1994.

[27] Pope C, Ziebland S, Mays N. Qualitative research in health care, Analysing qualitative data. BMJ 2000; 320: 114–6. 10625273 10.1136/bmj.320.7227.114 PMC1117368

[28] First MB, Gibbon M, Spitzer RL, Williams JBW, Benjamin LS. Structured Clinical Interview for DSM-IV Axis II Personality Disorders (SCID-II). American Psychiatric Press, 1997.

[29] Radcliffe J, Yeomans F. Transference-focused psychotherapy for patients with personality disorders: overview and case example with a focus on the use of contracting. Br J Psychother 2019; 35: 4–23.

[30] Paul M, Bluck G. Entering the world of psychotherapy: general psychiatric trainees take their first steps. Br J Psychother 1997; 14: 221–32.

[31] Robinson BL. Of mirrors, lamps and other methods for the writing up of notes. Br J Psychotherapy 2017; 33: 282–96.

[32] Davis JD, Elliott R, Davis ML, Binns M, Francis VM, Kelman JE, Development of a taxonomy of therapist difficulties: Initial report. Br J Med Psychol 1987; 60: 109–19. 3620387 10.1111/j.2044-8341.1987.tb02720.x

[33] Skovholt T, Rønnestad M. Struggles of the novice counselor and therapist. J Career Dev 2003; 30: 45–58.

[34] Rønnestad MH, Orlinsky DE. Clinical implications: training, supervision, and practice. In How Psychotherapists Develop: A Study of Therapeutic Work and Professional Growth (eds DE Orlinsky, MH Rønnestad): 181–202. American Psychological Association, 2009.

[35] Kächele H. Manualization as tool in psychodynamic psychotherapy research and clinical practice: commentary on six studies. Psychoanal Inq 2013; 33: 626–30.

[36] Swift J, Greenberg R. A treatment by disorder meta-analysis of drop-out from psychotherapy. J Psychother Integr 2014; 24: 193–201.

[37] Burbridge-James W, Husbands A. RCPsych Psychotherapy Survey: 2018 Training Report on 2016 Survey Data. RCPsych.

[38] Crits-Christoph P, Baranackie K, Kurcias J, Beck A, Carroll K, Perry K, Meta-analysis of therapist effects in psychotherapy outcome studies. Psychother Res 1991; 1: 81–91.

[39] Sathanandan S, Bull D. An exploration of core psychiatry trainees experience of and thoughts surrounding personal psychotherapy. Psychoanal Psychother 2013; 27: 77–82.

[40] Dover D, Beveridge E, Leavey G, King M. Personal psychotherapy in psychiatric training: study of four London training schemes. Psychiatr Bull 2009; 33: 433–436.

[41] Kernberg OF. Video illustration for Transference-Focused Psychotherapy for Borderline Personality Disorder: A Clinical Guide. American Psychiatric Publishing, 2020 (https://www.appi.org/yeomans [cited 11 Feb 2020]).

[42] Mura S, Ponsford H, Punter J, Sardana A, Sirohi S, Tanna J. Innovations in simulated and technology-aided learning for core psychiatric trainees in psychotherapy. Psychoanal Psychother 2013; 27: 83–7.

[43] Guest G, Namey E, McKenna K. How many focus groups are enough? Building an evidence base for nonprobability sample sizes. Field Methods 2017; 29: 3–22.

[44] Bateman A. Psychotherapy in psychiatry: integration and assimilation. Int Rev Psychiatry 2007; 19: 1–4. 17365153 10.1080/09540260601109448

